# Specific Immune Responses and Oncolytic Effects Induced by EBV LMP2A-Armed Modified Ankara-Vaccinia Virus Vectored Vaccines in Nasopharyngeal Cancer

**DOI:** 10.3390/pharmaceutics17010052

**Published:** 2025-01-03

**Authors:** Liying Sun, Chao Liu, Junping Peng

**Affiliations:** 1 NHC Key Laboratory of Systems Biology of Pathogens, Institute of Pathogen Biology, Chinese Academy of Medical Sciences, Peking Union Medical College, Beijing 100005, China; sunliying@ipbcams.ac.cn; 2 State Key Laboratory of Stress Biology, Fujian Provincial Key Laboratory of Innovative Drug Target Research, School of Pharmaceutical Sciences, Xiamen University, Xiamen 361102, China

**Keywords:** Epstein-Barr virus latent membrane protein 2A, modified vaccinia virus ankara, nasopharyngeal cancer, cytotoxic lymphocyte, anti-tumor effect

## Abstract

Background: The Epstein-Barr virus (EBV) is intricately linked to a range of human malignancies, with EBV latent membrane protein 2A (LMP2A) emerging as a potential target antigen for immunotherapeutic strategies in the treatment of nasopharyngeal carcinoma (NPC). Methods: The modified vaccinia virus Ankara (MVA) is universally used in vector vaccine research because of its excellent safety profile and highly efficient recombinant gene expression. Here, we constructed a novel MVA-LMP2A recombinant virus and investigated its specific immune response induction and oncolytic effect. Results: An immunization dose of 2 × 10^7^ PFU induced the highest specific immune response, which was no longer increased by boost injections after four doses. Three weeks post-final immunization, the specific immune response reached its peak. The MVA-LMP2A vaccine-induced LMP2A-specific cytotoxic T lymphocytes (CTLs), which exhibited substantial efficacy against target cells and effectively inhibited tumor growth. Conclusions: Thus, the MVA-LMP2A recombinant virus effectively induces strong LMP2A-specific cellular and humoral immune responses and anti-tumor activity. This work provides a promising therapeutic strategy for developing NPC candidate vaccines, as well as a reference for the treatment of EBV LMP2-associated malignancies.

## 1. Introduction

The Epstein-Barr virus (EBV) has a global prevalence exceeding 90%, with over 200,000 malignancy cases attributed to EBV annually [1]. EBV latent oncogenic infections are implicated in various human malignancies, such as nasopharyngeal carcinoma (NPC), gastric carcinoma, Hodgkin lymphoma (HL), post-transplant lymphoproliferative disorder (PTLD), and certain NK/T cell lymphomas [2,3]. NPC is particularly associated with EBV latent infection, characterized by the expression of key EBV proteins, including EBNA1, latent membrane protein (LMP)2A/B, and LMP1 [4]. LMP2A promotes epithelial-mesenchymal transition and proliferation of numerous nasopharyngeal carcinoma (NPC) spheroid cancer cells [5,6]. Early RT-PCR studies found that LMP2A mRNA was detected in >98% of patients with NPC, whereas LMP2B and LMP1 mRNA expression levels in these patients were very low [7,8]. Immunohistochemical results of NPC patients showed that LMP2A expression was significantly higher than 50%; thus, LMP2A represents a promising target antigen for eliciting Epstein-Barr virus (EBV)-specific immune responses [6].

Modified Vaccinia Virus Ankara (MVA) is an attenuated, replication-deficient variant of the vaccinia virus, possessing a structural configuration similar to that of the replicative vaccinia virus [9]. The absence of an effective viral replication region in MVA further enhances its safety profile as a vaccine vector [10]. The Modified Vaccinia Ankara (MVA) virus is unable to replicate in the majority of mammalian cells, yet it efficiently expresses recombinant genes, rendering it a highly effective platform for the delivery of exogenous genes [11]. When recombinant MVA is employed in vaccination, it successfully induces strong humoral and cellular immune responses as well as specific anti-tumor activity, particularly when transgenes are inserted into the thymidine kinase (TK) gene locus of the MVA [9,12,13,14,15].

Over the past two decades, substantial progress has been made in the multidisciplinary management of nasopharyngeal carcinoma (NPC), incorporating radiation therapy, combination chemotherapy, and surgical intervention, all of which have collectively contributed to a decline in mortality rates [16,17,18,19]. However, local treatment failure in aggressive cases of NPC remains common, and the occurrence of distant metastases by malignant tumor cells following surgical procedures persists as the leading cause of mortality. In such cases, immunotherapy directed at Epstein-Barr virus (EBV) antigens offers a promising and feasible therapeutic strategy. This study aimed to develop a recombinant vaccinia virus expressing the EBV LMP2A antigen (MVA-LMP2A) for potential applications in nasopharyngeal carcinoma (NPC) treatment. We evaluated the capacity of MVA-LMP2A to activate specific cytotoxic T lymphocytes in vitro to elicit robust and sustained EBV LMP2A-specific humoral and cellular immune responses, as well as to induce anti-tumor activity against target cells in vivo.

## 2. Materials and Methods

### 2.1. Cell Lines and Animals

BHK-21 hamster kidney fibroblasts were used for virus amplification, while TC-1-GLUC-LMP2 tumor cells expressing EBV LMP2 and Gaussia luciferase (GLUC) were used to assess anti-tumor efficacy [20]. Both cell types were cultured in Dulbecco’s modified Eagle’s medium (Corning, Manassas, VA, USA) with fetal bovine serum (Invitrogen, Middletown, VA, USA) and penicillin-streptomycin (Corning, Manassas, VA, USA).

Female BALB/c and C57BL/6 mice, aged 4 to 6 weeks, were procured from the Military Academy of Medical Sciences Animal Center and maintained under pathogen-free conditions at the China National Institute for Viral Disease Control and Prevention. The mice were anesthetized and subsequently euthanized via cervical dislocation.

### 2.2. Construction of MVA-LMP2A Vaccine

The MVA expressing LMP2A (NC_007605.1) recombinant virus was created by constructing the pZL-GFP-LMP2A shuttle plasmid, which includes parts of the vaccinia *TK* gene to enable homologous recombination. The plasmid was transfected into BHK-21 cells, which had been pre-infected with MVA for a duration of two hours, to generate the MVA-GFP-LMP2A recombinant virus, incorporating both *GFP* and *LMP2A* genes. Recombinant viruses were then selected by fluorescence microscopy. Following several rounds of selection, pure recombinant stock MVA-LMP2A expressing the exogenous gene for EBV LMP2A was obtained, and vaccinia viruses were purified via centrifugation over a sucrose gradient [21,22,23].

### 2.3. Polymerase Chain Reaction (PCR) and Western Blot

The MVA-LMP2A and MVA-NULL (MVA without LMP2A) viral genomes were extracted using Genomic DNA Mini kits (Qiagen, Düsseldorf, Germany). The LMP2A, TK, and GFP genes were amplified, and detection primers for LMP2A, TK, and GFP were designed (Table S1) to identify the LMP2A gene in the MVA-LMP2A vaccine.

Seventy-two hours after infecting BHK-21 cells with MVA-LMP2A or MVA-NULL, proteins were extracted using a Membrane Protein Extraction Kit and analyzed by SDS-PAGE. After transfer, membranes were incubated overnight with a rat anti-EBV LMP2-2A monoclonal antibody, followed by a one-hour incubation with an HRP-conjugated goat anti-rat IgG secondary antibody. Visualization was performed using a DAB kit.

### 2.4. Immunization Strategy

To evaluate LMP2A-specific immune responses to varying doses of MVA-LMP2A, 30 BALB/c mice were divided into five groups and intramuscularly immunized with 2 × 10^7^, 2 × 10^6^, 2 × 10^5^, or 2 × 10^4^ PFU of MVA-LMP2A in 100 µL PBS per mouse, with booster shots every two weeks. Control mice received MVA-NULL. One week after the last immunization, mouse splenic lymphocytes were collected for enzyme-linked immunosorbent spot (ELISPOT) detection of LMP2A-specific immune responses.

To evaluate the LMP2A-specific immune responses at various time points following the final immunization, 48 BALB/c mice were randomly allocated into eight groups. Each mouse received intramuscular immunization with 2 × 10^7^ plaque-forming units (PFU) of MVA-LMP2A suspended in 100 µL of phosphate-buffered saline (PBS), followed by single booster injections administered at two-week intervals. Control groups were immunized with MVA-NULL. Spleen lymphocytes were harvested from the mice at 0, 1, 2, 3, 4, 6, and 8 weeks post-final immunization, and an enzyme-linked immunospot (ELISPOT) assay was conducted to assess the LMP2A-specific immune responses.

To assess the specific immune responses at varying vaccination intervals, 42 BALB/c mice were randomly divided into seven groups. Each mouse received an intramuscular injection of 2 × 10^7^ PFU MVA-LMP2A suspended in 100 µL PBS. This initial immunization was followed by 0, 1, 2, 3, 4, or 5 booster injections administered at two-week intervals. One week subsequent to the final immunization, splenic lymphocytes were harvested from the mice for the purpose of assessing LMP2A-specific immune responses using the ELISPOT assay.

### 2.5. IFN-γ Detection by ELISPOT

To assess the specific immune responses at varying vaccination intervals, 42 BALB/c mice were randomly divided into seven groups. After 36 h of incubation, biotinylated secondary anti-IFN-γ antibody and streptavidin-ALP were added to each well. Spots were developed using a BCIP/NBT-plus substrate, and spot-forming cells were counted using an ELISPOT reader.

### 2.6. IFN-γ and TNF-α Detection by Flow Cytometry

Splenic lymphocytes from MVA-LMP2A-immunized mice were stimulated with anti-mouse CD28, an EBV LMP2-specific peptide, and IL-2, each at 10 μg/mL. Protease transport inhibitors brefeldin A and monensin sodium were also added, and the samples were incubated for 24 h. Negative control lymphocyte samples were prepared without LMP2 peptide stimulation. The splenic lymphocytes were washed with 2% fetal bovine serum in PBS and then stained for surface markers using fluorescently labeled monoclonal antibodies. The staining protocol involved adding 20 µL of four antibodies: V450-anti-mouse CD3, PerCP-Cy^TM^5.5-anti-mouse CD4, PE-anti-mouse CD8a, and APC-CD49b, all from eBioscience. Cells were permeabilized with a Cytofix/Cytoperm kit and stained with FITC-anti-mouse IFN-γ and APC-anti-mouse TNF-α, each at 2.5 μg/mL. Analysis was performed using all six color channels of the BD FACSCalibur flow cytometer(BD Biosciences, Heidelberg, Germany).

### 2.7. ELISA Detection of Anti-LMP2A Antibody

LMP2A-specific antibody titers were assessed using ELISA in the serum of mice immunized with 2 × 10^7^ PFU of MVA-LMP2A. Sera from MVA-NULL-immunized mice served as negative controls. The sera were diluted two-fold from 1:30 in 96-well ELISA plates coated with EBV particles and then treated with HRP-labeled goat anti-mouse IgG (10 μg/mL). The reaction was stopped with TMB substrate.

### 2.8. Cytotoxic Effect In Vitro

Lymphocytes from C57BL/6 mice immunized with either MVA-LMP2A or MVA-NULL were activated with IL-2 and used in serial dilutions as effector cells against TC-1-GLUC-LMP2 targets. The cell index was monitored using the iCELLigence system. L8 E-plates were incorporated into the iCELLigence system to monitor the cellular adherence rate over a period of 168 h. Subsequently, the data were exported for further analysis [20].

### 2.9. Tumor Challenge

Fourteen C57BL/6 mice were divided into two experimental groups: MVA-LMP2A and MVA-NULL. Each mouse was administered intramuscular immunization with 2 × 10^7^ plaque-forming units (PFU) of either MVA-LMP2A or MVA-NULL suspended in 100 μL of phosphate-buffered saline (PBS). This was followed by a booster injection two weeks later. Ten days subsequent to the initial immunization, the mice received a subcutaneous injection of 4 × 10^6^ TC-1-GLUC-LMP2 tumor cells in the groin region. The tumor-challenged mice were monitored and imaged on days 3, 7, and 14 using an in vivo imaging system (IVIS) chamber. Fourteen days post-tumor inoculation, spleen lymphocytes from C57BL/6 mice were harvested to evaluate the LMP2A-specific immune response via ELISPOT.

### 2.10. Statistical Analysis

Statistical analyses were performed utilizing *t*-tests and one-way ANOVAs, supplemented by chi-squared tests, through the application of GraphPad Prism version 7.0 (GraphPad Software Inc., San Diego, CA, USA). The values of the experimental groups were compared to those of the control group. A *p*-value of less than 0.05 was deemed indicative of statistical significance.

## 3. Results

### 3.1. The Overview of the Experimental Design

**Figure 1:**
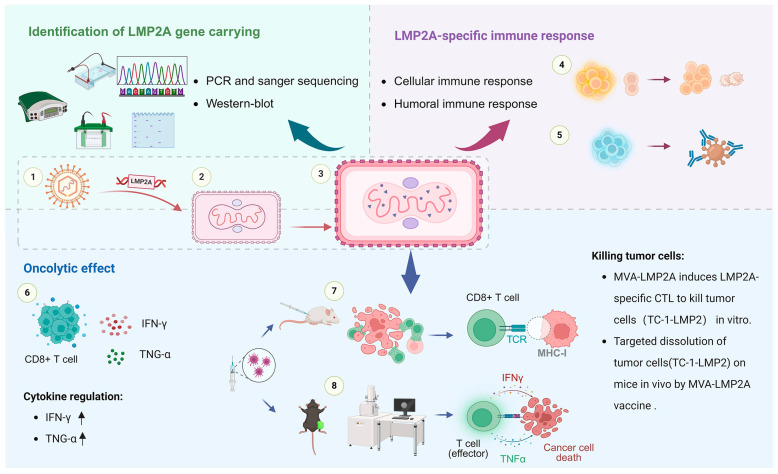
Schematic of the study design. Circle 1 indicates the Epstein-Barr virus (EBV) from which the LMP2A target gene was extracted. Circle 2 indicates the wild-type Ankara-vaccinia virus, which was used as the virus vector constructed in this study. Circle 3 indicates the combination virus that is MVA-LMP2A vaccine. Circle 4 indicates an activated LMP2A-specific cellular immune response. Circle 5 indicates activation of the LMP2A-specific humoral immune response. Circle 6 means the induced cytokine regulation. Circle 7 indicates the killing of tumor cells by MVA-LMP2A in vitro. Circle 8 indicates the killing of tumor cells by MVA-LMP2A in vivo. The vertical arrow signifies an increase in concentration.

This study was conducted in three primary phases: (1) identification of the gene and protein associated with MVA-LMP2A, (2) evaluation of humoral and cellular immune responses induced by MVA-LMP2A in mice, and (3) analysis of the oncolytic efficacy of the MVA-LMP2A vaccine (Figure 1).

### 3.2. The Construction of MVA-LMP2A Vaccine

**Figure 2:**
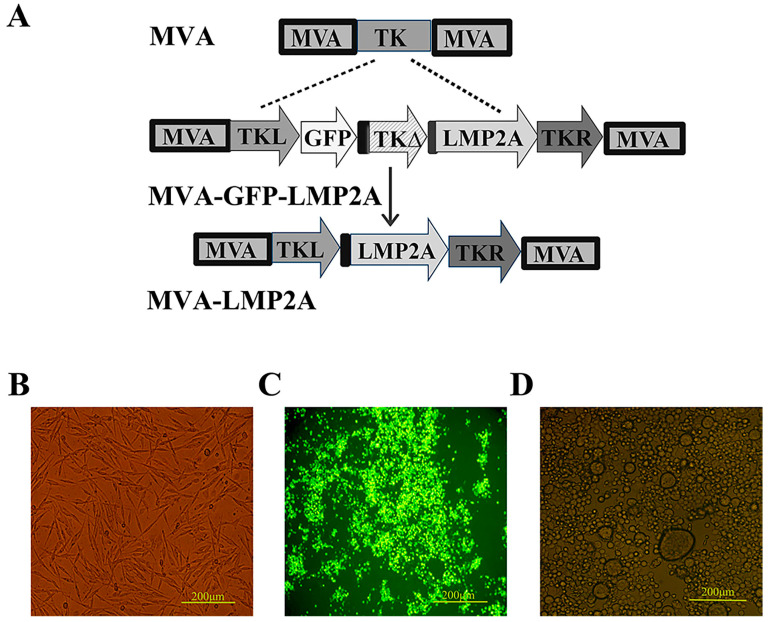
Presents the construction of MVA-LMP2A and corresponding microscopy images. (**A**) A schematic representation of the MVA-LMP2A construction process. (**B**–**D**) Microscopic observations of cells: (**B**) BHK-21 cells, (**C**) BHK-21 cells infected with MVA-GFP-LMP2A, and (**D**) BHK-21 cells infected with MVA-LMP2A. The dashed line illustrates the distinct genetic composition of the recombinant virus MVA-GFP-LMP2A, while the downward arrow denotes the specific genetic configuration of the recombinant virus MVA-LMP2A.

To validate MVA-LMP2A, we first constructed an MVA-GFP-LMP2A recombinant virus carrying the GFP and LMP2A genes by homologous recombination (Figure 2), and BHK-21 cells infected with this virus appeared green under a fluorescence microscope (Figure 2C). Using the self-deletion of homologous genes method and fluorescence microscopy, MVA-LMP2A cells expressing the LMP2A gene, but not the GFP gene, were selected. The MVA-LMP2A-infected cells became big and round, which are typical features of virus-infected cells, and did not appear green under fluorescence microscopy (Figure 2D).

### 3.3. The Polymerase Chain Reaction (PCR) and Western Blot Identification of the EBV LMP2

**Figure 3:**
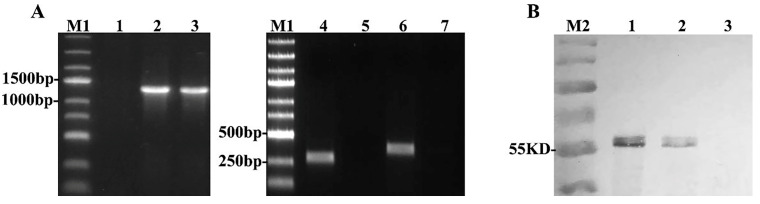
PCR and western blot detection of LMP2A. (**A**) PCR detection of the LMP2A gene. Lane M1: DNA ladder; lane 1: LMP2A primer-amplified MVA-NULL genome fragment; lane 2: LMP2A primer-amplified MVA-LMP2A genome fragment; lane 3: LMP2A primer-amplified pZL-GFP-LMP2A plasmid fragment; lane 4: TK primer-amplified MVA-NULL fragment; lane 5: TK primer-amplified MVA-LMP2A fragment; lane 6: GFP primer-amplified pZL-GFP-LMP2A plasmid fragment; lane 7: GFP primer-amplified MVA-LMP2A fragment. (**B**) Western blot detection of LMP2A protein. Lane M2: PageRuler Prestained Protein Ladder; lane 1: MVA-LMP2A-infected BHK-21 cells; lane 2: BHK-21 cells transfected with pZL-GFP-LMP2; lane 3: MVA-NULL-infected BHK-21 cells.

To confirm the successful construction of MVA-LMP2A, LMP2A, TK, and GFP genes were measured by PCR. The results verify that MVA-LMP2A carried the LMP2A gene, and the fragment was 1494 bp. The TK primers did not produce any amplification products, indicating that the exogenous gene was integrated into the TK region of MVA. Consequently, due to the self-deletion of homologous genes, amplification of the GFP gene also yielded negative results (Figure 3A). Together, these results indicate that a pure MVA-LMP2A recombinant virus expressing only the LMP2A gene was obtained.

Western blot analyses were conducted to detect LMP2A protein in BHK-21 cells infected with MVA-LMP2A, BHK-21 cells transfected with pZL-GFP-LMP2, and untransfected BHK-21 cells. The findings demonstrate that the molecular weight of LMP2A is 55 kDa, consistent with the positive control, pZL-GFP-LMP2A (Figure 3B), thereby confirming that MVA-LMP2A effectively expresses the LMP2A protein.

### 3.4. The Specific Immune Responses Induction of MVA-LMP2A Vaccine

**Figure 4:**
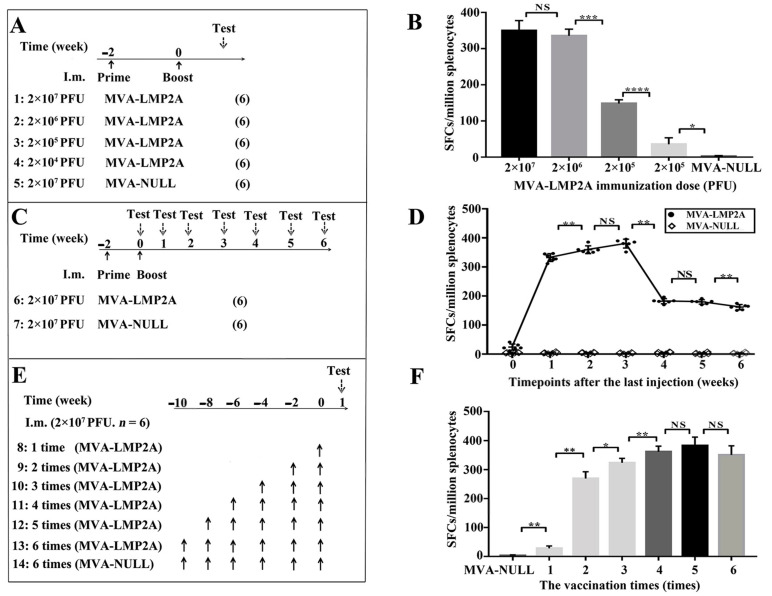
The immunization strategies and MVA-LMP2A-induced specific immune responses. (**A**) The immunization strategy for MVA-LMP2A injection at diverse doses. (**B**) The immune response effects of MVA-LMP2A injection at diverse doses. (**C**) The immunization strategy for different timepoints after the last inoculation. (**D**) The immune effects detected at different timepoints after the last inoculation. (**E**) The immunization strategy for various vaccination times. (**F**) The LMP2A-specific immune responses were analyzed at various vaccination times. * *p* < 0.05; ** *p* < 0.01; *** *p* < 0.005; **** *p* < 0.001; NS, no significant difference; the data represents mean ± s.d. (*n* = 6) in (**B**,**D**,**F**). (6) representing 6 mice in each group. the upward arrow denotes the point of vaccination, while the downward arrow indicates the time of analysis following the final immunization. I.m. stands for intramuscular injection.

This study aimed to examine the in vivo effects of various vaccination strategies on the LMP2-specific immune response. Mice were administered varying doses of MVA-LMP2A, and the resultant LMP2A-specific immune response was assessed one week following the final immunization (Figure 4A). The ELISPOT results show that an LMP2A-specific immune response was induced in mice immunized with 2 × 10^4^ PFU, and the number of LMP2A-specific spot-forming cells (SFCs) increased in an immunization dose-dependent manner. The highest specific immune response (350 SFCs) was induced by immunization with 2 × 10^7^ PFU MVA-LMP2A (Figure 4B).

BALB/c mice received two immunizations of 2 × 10^7^ PFU of MVA-LMP2A at two-week intervals, and the impact on the LMP2A-specific immune response was evaluated at multiple time points post-final immunization (Figure 4C). The ELISPOT results showed that the LMP2A-specific immune response gradually increased with prolonged immunization times, reaching a peak immune response 3 weeks after the last immunization. After this time, the number of LMP2A-specific SFCs significantly decreased, although some still existed 8 weeks after the last immunization (Figure 4D).

BALB/c mice received 2 × 10^7^ PFU of MVA-LMP2A at different intervals, and their immune response was assessed a week after the last dose (Figure 4E). The response improved with each dose but plateaued after three boosters, with no significant increase in the LMP2A-specific SFCs after four doses (Figure 4F).

### 3.5. The Specific IFN-γ and TNF-α Analysis Induced by EBV-LMP2A Vaccine

**Figure 5:**
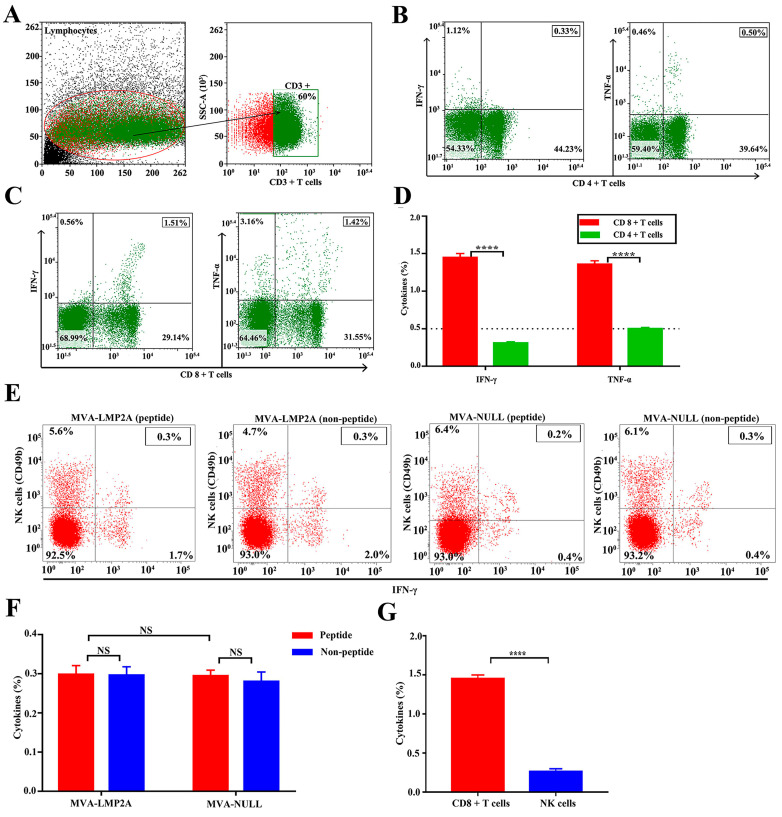
Presents the results obtained from flow cytometry analysis. (**A**) illustrates the gating strategy for CD3+ T cells using flow cytometry. (**B**,**C**) display the levels of IFN-γ and TNF-α in CD4+ and CD8+ T cells, respectively. (**D**) shows the cytokine percentages in both CD4+ and CD8+ T cells. (**E**) depicts IFN-γ levels in NK cells following exposure to the LMP2-peptide derived from MVA-LMP2A and MVA-NULL mice, as well as in the absence of the LMP2-peptide. (**F**) presents the percentages of IFN-γ in NK cells, while (**G**) illustrates the percentages of IFN-γ in both CD8+ T and NK cells. The experiment was conducted twice, with columns representing the mean ± standard deviation (n = 5) in (**D**,**F**,**G**). Statistical significance is indicated by **** *p* < 0.001, while NS denotes no significant difference. Red represents polypeptide stimulation, and blue represents no polypeptide stimulation.

In this study, our objective was to delineate the cellular immune responses specific to LMP2A induced by the MVA-LMP2A vaccine. We focused on quantifying the production of interferon-gamma (IFN-γ) in CD4+ and CD8+ T lymphocytes post-immunization. BALB/c mice were initially inoculated with 2 × 10^7^ plaque-forming units (PFU) of MVA-LMP2A, followed by a booster injection two weeks later. One week after the final immunization, splenocytes were extracted from the mice for subsequent analysis using flow cytometry. CD3+ T cells were isolated and characterized utilizing flow cytometry techniques, as depicted in Figure 5A. The proportions of IFN-γ and TNF-α within CD4+ and CD8+ T cells were evaluated through flow cytometry analysis. The results demonstrate that IFN-γ and TNF-α levels were significantly higher in CD8+ T cells compared to CD4+ T cells (Figure 5B,C). These findings suggest that the induction of LMP2A-specific immune responses by MVA-LMP2A is primarily dependent on CD8+ T cells (Figure 5D).

Meanwhile, we analyzed the IFN-γ levels in NK cells induced by MVA-LMP2A. Splenocytes from mice vaccinated with MVA-LMP2A or MVA-NULL were examined by flow cytometry, with a negative control lacking LMP2-peptide stimulation. CD49b was used to assess IFN-γ levels in NK cells. The results indicated that IFN-γ release in NK cells from mice immunized with MVA-LMP2A was minimal, showing no difference from controls without the LMP2-peptide or those from MVA-NULL (Figure 5E,F). Additionally, IFN-γ levels in CD8+ T cells were significantly higher than those in NK cells (Figure 5G), confirming that the LMP2-specific immune response induced by MVA-LMP2A was primarily dependent on CD8+ T cells.

### 3.6. The Specific EBV LMP2A Antibody Detected by Enzyme-Linked Immunosorbent Assay (ELISA)

**Figure 6:**
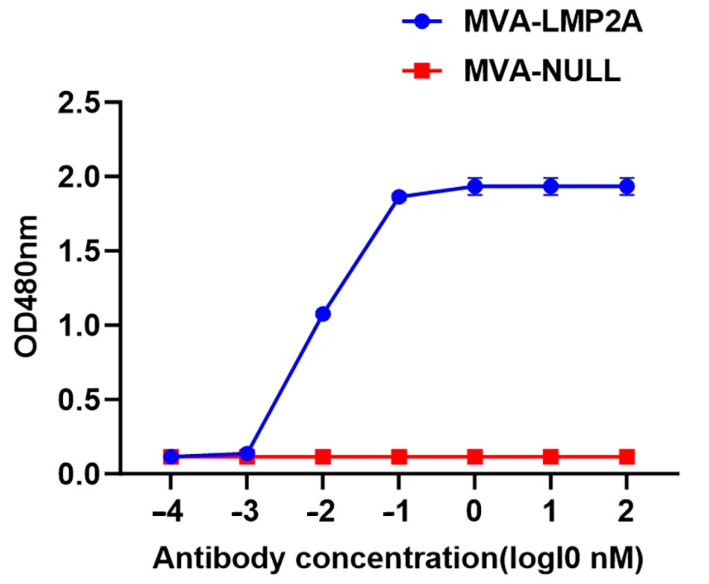
EBV LMP2A-specific antibody test results. The blue line represents the antibody concentration of MVA-LMP2A, and the red line represents the antibody concentration of MVA-NULL.

Evaluation of the LMP2A-specific humoral immune response elicited by MVA-LMP2A. Serum samples were collected from mice immunized with MVA-LMP2A and subjected to serial two-fold dilutions, ranging from 1:100 to 1:10,000. The diluted sera were then applied to 96-well plates coated with EBV antigens for ELISA analysis. The cutoff value was 2.5 times the OD value of MVA-NULL mice sera. The lowest dilution of MVA-LMP2A serum with detectable LMP2A-specific antibody was 100–1000 fold (Figure 6). These results indicate that MVA-LMP2A significantly induces a specific humoral immune response.

### 3.7. Detection of EBV-LMP2A Specific Cytotoxic Effect in Vitro

**Figure 7:**
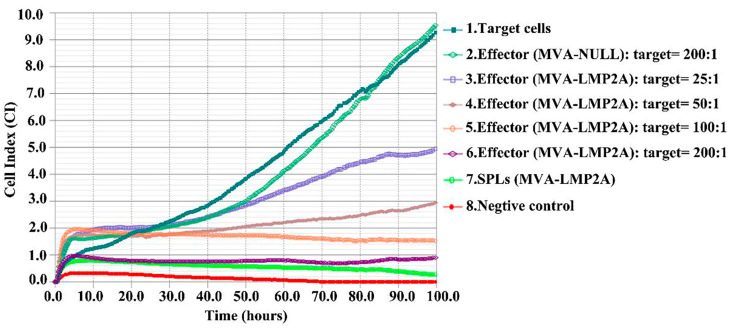
The cytotoxic T lymphocyte (CTL) activity specific to LMP2A in the elimination of target cells. Line 1 represents 2 × 10^3^ TC-1-GLUC-LMP2 target cells, while Line 2 depicts a mixture of 4 × 10^5^ spleen cells (SPLs) induced by MVA-NULL; Line 3: 5 × 10^4^ MVA-LMP2A-induced SPLs mixed cells; Line 4: 1 × 10^5^ MVA-LMP2A-induced SPLs mixed cells. Line 5: 2 × 10^5^ MVA-LMP2A-induced SPLs mixed cells; Line 6: 4 × 10^5^ MVA-LMP2A-induced SPLs mixed cells; Line 7: 4 × 10^5^ MVA-LMP2A-induced SPLs; Line 8: Roswell Park Memorial Institute (RPMI) 1640.

To assess LMP2A-specific oncolytic activity in vitro, splenic lymphocytes were diluted and mixed with TC-1-GLUC-LMP2 target cells. Sixteen hours later, CTLs began to lyse the target cells. The efficacy of the specific cytotoxic effect was significantly augmented with increasing concentrations of LMP2A-based CTLs, as illustrated in Figure 7, lines 3–6. The cell index (CI) of the 4 × 10^5^ spleen lymphocytes (SPLs) mixed group (Figure 7, line 6) was very similar to that of SPLs (Figure 7, line 7), indicating the effective killing ability of MVA-LMP2A-induced CTL. Because SPLs were collected from MVA-NULL-immunized mice without oncolytic potency, the index line of the MVA-NULL group (Figure 7, line 2) was almost the same as that of the target cells (Figure 7, line 1). Together, these data suggest that MVA-LMP2A-induced specific CTL possess a significant capability to kill target cells in vitro.

### 3.8. The Oncolytic and Anti-Tumor Effect of MVA-LMP2A Vaccine

**Figure 8:**
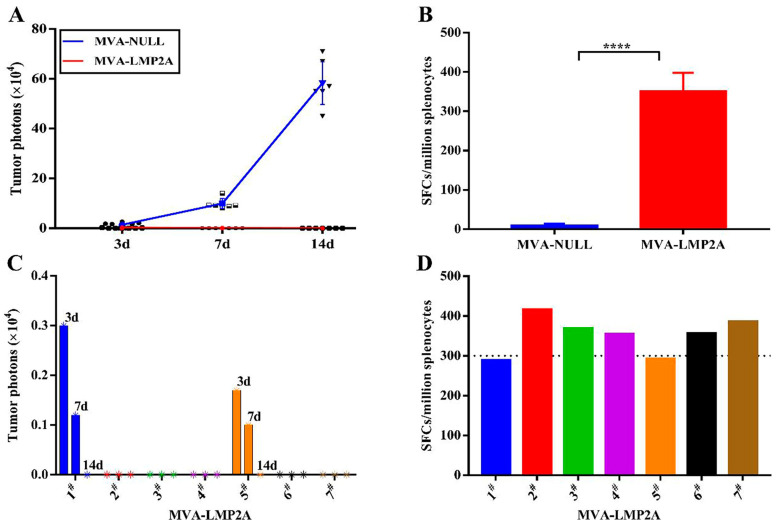
Shows the oncolytic effect of the MVA-LMP2A vaccine. (**A**) tumor photon counts in mice were measured using an IVIS imaging system; (**B**) LMP2-specific immune responses were assessed with an ELISPOT assay; (**C**) tumor photon counts in seven MVA-LMP2A-inoculated mice were analyzed by IVIS. (**D**) The count of LMP2-specific SFCs in seven MVA-LMP2A-immunized mice is shown. **** *p* < 0.0001; data is mean ± s.d. in (**A**,**B**), (*n* = 7).

This study evaluated the anti-tumor effects of MVA-LMP2A in vivo. Ten days after the final immunization with either MVA-LMP2A or MVA-NULL, mice were injected with TC-1-GLUC-LMP2 tumor cells. Tumor development was monitored and imaged in vivo on days 3, 7, and 14 post-inoculation. The LMP2A-specific immune response was evaluated on day 14. In MVA-NULL-immunized mice, the quantity of tumor-associated photons exhibited a gradual increase corresponding to the duration of tumor cell inoculation. In contrast, no photons were detected in mice vaccinated with MVA-LMP2A (Figure 8A). Furthermore, ELISPOT assay results demonstrated that mice immunized with MVA-LMP2A exhibited significantly elevated LMP2A-specific immune responses compared to those injected with MVA-NULL (Figure 8B, n = 7, *p* < 0.0001). In the MVA-LMP2A inoculation group, complete tumor regression was observed in five mice by the third day following tumor cell injection (Figure 8C). Additionally, by the fourteenth day post-injection, all seven mice immunized with MVA-LMP2A exhibited effective tumor cell clearance. ELISPOT analysis further indicates that these five mice generated a significantly higher number of LMP2-specific spot-forming cells (SFCs) compared to the remaining two mice in the group (Figure 8D). Together, these results indicate that MVA-LMP2A effectively inhibits tumor growth and clears tumor cells in vivo, and the anti-tumor effect gradually improves with increases in the LMP2A-specific immune response.

## 4. Discussion

Epstein-Barr virus (EBV)-related cancers are treated using chemotherapy, immunotherapy, and targeted viral therapy. Among immunotherapies, the adoptive transfer of EBV-specific cytotoxic T lymphocytes (EBV-CTLs) effectively eliminates EBV-infected cells and is used for post-transplant lymphoproliferative disorder (PTLD), nasopharyngeal carcinoma (NPC), and Hodgkin lymphoma (HL) [18,24,25]. This treatment prevents further development of PTLD; however, its therapeutic effect in NPC and HL patients is less clear [26,27,28]. Among NPC patients treated with standard radiotherapy and chemotherapy, 15–19% have tumor cell metastasis, and locally severe patients have poor survival [29]. In such cases, immunotherapy is another treatment option. The application of immunotherapy has generally focused on patients with recurrent NPC; it can successfully prevent relapses, lacks obvious side effects, and prevents further development of disease in some patients [30,31]. EBV LMP2A is a promising target antigen for EBV-CTLs, and administration of this protein induces efficient LMP2A-specific immune responses when it is used for NPC immunotherapy [6,32,33]. However, data regarding this type of immunotherapy are currently very limited, so further research on the immunotherapy of EBV-related tumors is still needed. Additionally, the establishment of EBV-related vaccinations will play an important role in malignancy prevention and public health [34,35].

Viral vectors have been extensively utilized as a potent technological approach for combating various diseases, including malaria, tuberculosis, influenza, AIDS, hepatitis C, and Ebola [36]. The repertoire of application vectors encompasses adenovirus, herpes virus, yellow fever virus, and alphavirus [37,38,39,40]. Phase I clinical trials involving an HIV adenovirus vector vaccine demonstrated that it elicited robust HIV-specific CD4+ and CD8+ T cell immune responses in the majority of participants, with peak cellular immunity observed approximately four weeks following the final immunization [41]. However, the presence of adenovirus 5 (Ad5)-neutralizing antibodies in adults limits the application of adenovirus vectors as human vaccines, and studies in non-primates have also reported that previous immunization with Ad5 inhibited cellular immune responses to Ad5-vectored vaccines [42,43]. Here, our results in immunized mice similarly show that 2 × 10^7^ PFU of MVA-LMP2A significantly induced LMP2A-specific immune responses, which reached peak levels at 3 weeks after MVA-LMP2A immunization. The EBV-specific SFCs no longer increased after four administrations of the recombinant virus, suggesting that the mice may produce neutralizing antibodies against MVA, limiting the improvement in LMP2A-specific immune responses. However, among the four vaccine administrations, the LMP2A-specific cellular immune response increased significantly as the number of vaccinations increased.

NPC has dense lymphoid tissue infiltration [44]. CD3 is an important lymphocyte marker, and the main surface markers of helper T lymphocytes and cytotoxic T lymphocytes are CD4 and CD8, respectively. Therefore, we performed flow cytometry using these markers to investigate cytotoxic T cells capable of killing tumor cells [45]. Our research supports the idea that vaccine-induced effector memory CD8+ T cells may be an important T cell subset for stimulating an effective therapeutic vaccine against tumors. This study provides evidence that T cell cytokine-secretion responses induced by MVA-LMP2A are mainly mediated by eliciting a high frequency of antigen-specific CD8+ T cells co-expressing IFN-γ/TNF-α.

A Phase I clinical trial investigating MVA-EL, an MVA recombinant virus armed with EBNA1/LMP2, demonstrated that all 16 patients in the six high-dose vaccination cohorts exhibited significant EBNA1/LMP2-specific immune responses without experiencing adverse events. These findings suggest that the MVA-vectored vaccine possesses both effective safety and immunogenicity [46]. Furthermore, many studies have shown that MVA-related vectored vaccines significantly induce specific cellular immune responses and effectively kill tumor cells [9,11,47]. Similarly, our results indicate that the tumors disappeared by 3 days post-tumor cell injection in five of the seven MVA-LMP2A-immunized mice, and they were completely eliminated by 14 days post-target cell inoculation in all MVA-LMP2A-injected mice. The five mice with faster tumor elimination had stronger LMP2A-specific immune responses than the other two, indicating a link between this immune response and oncolytic ability. As the EBV-specific immune response strengthened, tumor cells were destroyed more quickly and effectively.

In summary, we successfully constructed an MVA-LMP2A recombinant virus that effectively expresses the EBV LMP2A protein. Additionally, we demonstrated that intramuscular injections of MVA-LMP2A induced strong and long-lasting EBV LMP2A-specific cellular and humoral immune responses, effectively killed tumor cells, inhibited tumor growth, and stimulated a potent anti-tumor response in a murine tumor model. In future studies, we will assess the safety profile and potential side effects of this vaccine. Additionally, as part of our evaluation of this candidate vaccine, we will investigate its efficacy in combination with other vaccines, such as pCDNA3.1 and adenovirus-loaded vaccines, to explore the effectiveness of a joint immunization strategy. Furthermore, we will conduct an in-depth analysis of the specific anti-tumor mechanisms and cytotoxic effects associated with the vaccine. Collectively, our study expands our understanding of EBV LMP2A-targeting for an oncolytic effect and provides a promising vaccine candidate for the treatment of EBV-related malignancies.

## Data Availability

The data that support the findings of this study are available from the corresponding author upon reasonable request.

## Supplementary Materials

The following supporting information can be downloaded at: https://www.mdpi.com/article/10.3390/pharmaceutics17010052/s1, Table S1: title, Primers for detection of MVA-LMP2A virus vaccine.
