# Supplementary material for: Specific Immune Responses and Oncolytic Effects Induced by EBV LMP2A-Armed Modified Ankara-Vaccinia Virus Vectored Vaccines in Nasopharyngeal Cancer
Source: Pharmaceutics. 2025 Jan 3;17(1):52. doi: 10.3390/pharmaceutics17010052 (PMC11768126; doi:10.3390/pharmaceutics17010052)
Supplement: Supplementary file 1 [file pharmaceutics-17-00052-s001.zip › pharmaceutics-3326125-supplementary.pdf]

Article

Specific immune responses and Oncolytic effects induced by EBV LMP2A-armed modified Ankara-vaccinia virus vectored vaccines in Nasopharyngeal Cancer

Liying Sun [1], Chao Liu [2,*] and Junping Peng [1,*]

[1] NHC Key Laboratory of Systems Biology of Pathogens, Institute of Pathogen Biology, Chinese Academy of Medical Sciences & Peking Union Medical College, Beijing, China; pengjp@hotmail.com
[2] State Key Laboratory of Stress Biology and Fujian Provincial Key Laboratory of Innovative Drug Target Research, School of Pharmaceutical Sciences, Xiamen University; liuchao66888@xmu.edu.cn
* Correspondence: pengjp@hotmail.com; liuchao66888@xmu.edu.

Table S1 Primers for detection of MVA-LMP2A virus vaccine

Gene name	Primers
LMP2A	(+)GCTGCAGGAAACAACTCCCAATATCCA
	(-)AACTGGAGGGCAGCATCTAATGACC
TK	(+)CGGGACTATGGACGCATG
	(-)CGGTTTCCTCACCCAATCG
GFP	(+)CTCCAGCAGGACCATGTG
	(-)GGACGACGGCAACTACAAG

(+): forward primers; (-): reverse primers.
